# The Global Evolution of Travel Visa Regimes

**DOI:** 10.1111/padr.12166

**Published:** 2018-08-23

**Authors:** Mathias Czaika, Hein de Haas, María Villares‐Varela

## Introduction

SCHOLARS HAVE ARGUED that mobility is one of the key processes of globalization (Urry 2002; Sassen 2000; Elliot and Urry 2010) and is connected to the increase in international travel. As travelers can witness at international airports, citizenship and identification papers have a great influence on the ease of travel. While people holding passports of the wealthy or befriended countries can often breeze through custom checks, often using fully automated systems such as “e‐gates,” citizens of poor and politically fragile countries, particularly from South and South‐East Asia, the Middle East and Africa, generally require a travel visa to enter and have to queue up in long rows at the border. This seems to mirror a structural inequality in immigration and travel rights: travel visas are generally required for citizens of countries in the “Global South” who are often perceived by government officials and policy makers in the “Global North” as an immigration risk in terms of their potential to seek asylum seekers or to overstay their visas, or perceived as a security risk in terms of their potential to threaten public life as criminals or terrorists.

However, the perception of a North‐South mobility divide may reflect a Western or Eurocentric perspective and a bias created by the fact that Westerners mainly travel through Western airports where they may face only minor travel constraints. Realities seem to be more complex than this dichotomous representation. For instance, European citizens or North Americans travelling to sub‐Saharan Africa and countries including Russia, China, and India often need visas to do so and may find themselves queuing at borders too. While citizens of regional blocs such as the Economic Community of West African States (ECOWAS), the European Union (EU), the Gulf Cooperation Council (GCC) and the South African Development Community (SADC) enjoy free intra‐regional travel, travelers from Europe and other third countries may well need an entry visa. This suggests that real‐world travel visa regimes may not fit within simplistic North‐South schemes and that visa regimes may partly reflect more complex geopolitical relations and multi‐layered hierarchies at the regional level. For instance, Morocco grants visa‐free travel to citizens of particular, befriended states in Africa, such as Senegal and Mali, as well as to EU member states and the United States, but denies it to many fellow African and Arab citizens. Often, travel visa regimes are a negotiation chip in broader geopolitical games. For instance, the prospective lifting of EU visas for Turkish citizens was an important element of the March 2016 deal struck between the EU and Turkey in the context of the refugee crisis, as discussed further below.

This challenges the idea that we live in times of an increasing global mobility divide (e.g., Mau et al. 2015). In their analysis of visa waiver policies of over 150 countries for 1969 and 2010, those researchers found that while, at a global level, visa‐free mobility has increased, the inequality in visa waivers has also increased. They argue that, while citizens of wealthy countries, often members of the industrial‐country Organization for Economic Cooperation and Development (OECD) have gained mobility rights, those rights for other regions have stagnated or even diminished—in particular for citizens from African countries. They referred to this global bifurcation in mobility rights as the global mobility divide.

While their analysis shows the considerable potential of visa data as a means by which to study people's mobility rights and as a proxy of migration policies, there are also a number of limitations to their approach. First, while the notion of a global mobility divide seems a useful concept to describe the global pattern, this may conceal underlying complexities and patterns of “multi‐polarity,” for instance through the emergence of regional free‐mobility blocs such as ECOWAS or the EU. Second, a one‐sided focus on North‐South visa requirements is also likely to obscure considerable differences across countries within these two imaginary blocs, both in terms of the ability of their own citizens to travel freely to other countries (outbound restrictiveness) as well as the ability of foreign citizens to enter their territory (inbound restrictiveness). Third, if we wish to gain a comprehensive picture of mobility rights, we should look not only at entry requirements but also at exit requirements in the form of the limitations several governments have historically imposed on the international mobility of their own citizens. Fourth, to understand the nature and evolution of international visa regimes, we need to study how visa requirements are embedded in broader international relations and power asymmetries by assessing the bilateral (and inter‐regional) reciprocity of visa regimes. Fifth, to identify intertemporal shifts in visa regimes and foreign policy priorities, we require longitudinal bilateral panel data on travel visa restrictions over long periods, which have not been available so far.

To build upon the work by Mau et al. (2015), and further advance insights into the evolution and nature of global migration regimes, this paper aims to fill those gaps by exploring the purposes, patterns, and trends of bilateral visa policies from 1973 to 2013, with a particular focus on the evolution of patterns of visa restrictiveness, and bilateral and intra‐regional reciprocity. The analyses draw on the innovative global DEMIG VISA database, a new panel database that tracks annual bilateral travel visa requirements for 214 of 237 countries and territories over the 1973–2013 period. DEMIG VISA captures both entry visa and exit permit requirements according to the data reported in the Travel Information Manuals published monthly by the International Air Transport Association (IATA 1973–2013).^1^

The DEMIG VISA database provides a unique way to measure the evolution of migration policy. While travel visas are a suitable proxy to measure the evolution of migration policies (Salter 2006; Neumayer 2006; Finotelli and Sciortino 2013; Czaika and Trauner 2018), travel visa data can also be a useful vantage point to study patterns of regional integration (Gülzau et al. 2016) and international power relations more broadly.

By constructing indices of cross‐regional inbound and outbound entry as well as exit restrictiveness, we investigate: (1) the extent to which different world regions and regional unions have opened or closed vis‐à‐vis other regions; (2) the ways in which the formation of regional unions or the disintegration of countries or unions of countries (e.g., the USSR) has affected international visa regimes, and (3) the degree of bilateral reciprocity or policy asymmetry of visa regimes, which reflects the extent to which the introduction (or lifting) of a bilateral visa requirement by one country is mirrored by a retaliating introduction (or gratifying lifting) of visa requirements. This allows us to analyze the extent to which we can speak of a growing global mobility divide, or whether this typology adequately reflects real‐world patterns.

## Travel visas as a population and migration control instrument

Travel visas, alongside passports, have been key instruments of population movement control by modern states (Salter 2006; Finotelli and Sciortino 2013; Czaika and Trauner 2018). Modern state formation coincided with an increasing desire to monitor and control population mobility, both within and across borders. Until the twentieth century, states were generally more preoccupied with preventing exit rather than controlling immigration (Torpey 1998). From the sixteenth to the eighteenth century, emerging mercantilist states in northern Europe saw the population as a valuable economic resource and as potential recruits for the military. At the same time, state‐building ideals favored conformity, which pushed states to pursue ethnic and cultural homogeneity. This was implemented partly through the creation of new national foundation myths and unitary languages propagated through the education system as well as the circulation and mixing of military personnel, police, and other state employees such as teachers, and sometimes through the marginalization, murder, or expulsion of people who were physically or culturally different (Zolberg 1978; Anderson 1983).

Torpey (1998) argued that, as centralized national governments consolidated in the nineteenth century, they acquired the right to control the movement of their citizens. This control was reinforced by their ability to grant permission to leave based on the issuance of identity documents, including passports. It is the increased administrative capacity of modern states to maintain written records of populations in order to exert surveillance (Foucault 1975) that has given states the power to grant their citizens permission to leave based on passport issuance and, thus, to control movement. However, the spread of modern capitalism, accelerating population growth, widespread urban poverty, as well as industrialization and advances in military technology—which reduced the importance of population size for economic and military power—decreased the importance of population size and brought more positive attitudes towards emigration. This sparked the so‐called exit revolution (Zolberg 2007) during the late nineteenth and early twentieth century, in which states increasingly switched from emigration to immigration control. In this context, travel visas have become an essential instrument for migration control. Although visa regimes primarily regulate the entry of tourists and other temporary visitors, in practice they also function as instruments of migration control. In recent decades, travel visa regulations have often been used to prevent the entry of potential asylum seekers and visa overstayers, the latter generally seen as a more important source of unauthorized stay than unauthorized entry (Schoorl et al. 2000).

To complement travel visas, destination countries introduced carrier sanctions in the 1980s and 1990s to prevent people without a visa from boarding airplanes in the first place. So, combined with carrier sanctions, travel visas have become an important extraterritorial policy instrument to prevent people from entering and asking for asylum (Neumayer 2006). The travel visa should, therefore, be seen as a key component of the migration policy toolbox. For states, visa restrictions are considered an efficient, upfront way of preventing undesirable migrants from entering the national territory. This seems particularly effective for geographically‐distant origin countries that are reachable only by air, since this allows focusing immigration controls on a few ports of entry, instead of surveillance of long land and sea borders. What makes visas even more attractive is that they can generally be imposed through directives, decrees, or other administrative measures, and generally do not require cumbersome legal changes and, hence, parliamentary and judiciary procedures. They can, therefore, be implemented rather quickly, and discretely, by imposing restrictions on selected nationalities (Czaika and de Haas 2016).

In addition to crucial instruments used by states to control and monitor the entry of foreign visitors and migrants and the exit of nationals, travel visas also have an important symbolic function. After all, visas are a powerful expression of modern states’ sovereignty in monopolizing the “legitimate means of movement” (Torpey 1998). This usually pertains to the sovereignty states have in limiting the movement of their own citizens (either internally or abroad) or in controlling the immigration of foreign citizens. Travel visas are visible expressions of this power, as can be witnessed by travelers at airports and other border crossing points. Besides being an effective instrument for regulating entry and managing population mobility generally, the highly visible prioritization of own‐citizens and citizens of befriended nations and the concomitant discrimination vis‐à‐vis travelers in need of a visa (who usually have to queue for longer and are subject to more stringent checks) lend to border controls an important performative, symbolic dimension. These checks are one of the most visible means of migration control, through which states exert their sovereignty and reassure citizens that borders are indeed under control. US President Trump's 2017 ban on immigration from seven predominantly Muslim countries illustrates the ways in which travel visa regulations can be used as a convenient means by which to back up migration rhetoric and regulations. This is particularly the case when compared with other legislative instruments such as migration acts, since travel visa regulations often fall under executive powers which do not require legal changes, thus allowing governments to make changes while avoiding lengthy parliamentary debates. Moreover, their effects transcend implications for migration control but also shape the direction of foreign policy and international relations more broadly (Foreign Policy 2017). Policy reactions from those countries targeted by the recent US ban reflect how the introduction of visa requirements (in this case for US citizens entering these countries) are potentially used as retaliatory measures to challenge power asymmetries (Independent 2017; Al Jazeera 2017). Such practices to regulate mobility and the underpinning intricate power relations are at the core of this paper.

Visa regimes are thus a key expression of the ways in which states monopolize the legitimate means of movement (see Torpey 1998). Because of their relation to the crossing of state borders, this also implies that visa policies cannot be seen in isolation from international relations. As Finotelli and Sciortino (2013: 97) point out for the EU visa system, “the prevention of irregular migration is only one state interest out of many […] it must also be acknowledged that, in the creation of the EU visa regime, geopolitical considerations have often played a liberalizing role.” After all, the imposition of immigration controls implies an infringement of the liberties enjoyed by citizens of other states. In the first place, this helps us to explain why exit restrictions are increasingly seen as untenable in a context of increasing emphasis on human rights, which first and foremost apply to a country's own citizens. For states that perceive themselves to be democratic, or like to portray themselves as such, infringing the mobility rights of their own people, either through controlling internal mobility—such as in the Chinese *hukou* system (Liu 2005), or through exit visas, such as was for instance common in Communist and Fascist states—has become increasingly unacceptable. This kind of restriction is seen more and more as not compatible with observation of fundamental human rights—as stated in article 13 of the Universal Declaration of Human Rights (United Nations 1948). Yet, the granting of greater mobility rights to citizens has coincided with continued legitimation of subjecting foreign citizens to entry visa requirements at the will of the state. Travel visa regulations are legitimated by the virtue of modern citizenship: we can see visa regimes as systems of institutionalized discrimination based on nationality, which can be justified from the logic of protecting the exclusive residency and political rights of citizens.

## Travel visas as a foreign policy instrument

As discussed above, visa regimes should also be seen as an inherent part of foreign policy, as the curtailing or lifting of entry restrictions for citizens of particular countries is also a political statement and can therefore only be understood by taking into consideration the broader context of bilateral or multilateral relations. In this context, we distinguish bilateral and multilateral travel visa dynamics or interactions. The first is visa retaliation, in which the introduction of a visa by country X for citizens of country Y leads to punitive, antagonistic retaliation by the reciprocal introduction of visa requirements for citizens by country Y for citizens of country X. The unilateral imposition of visa regimes can be seen as a diplomatic affront, particularly when this happens outside of the context of broader diplomatic deals. For instance, when the Netherlands introduced a visa for Moroccan citizens in 1984, the Moroccan government took offense and introduced visa requirements for Dutch citizens wishing to travel to Morocco (DEMIG VISA 2015).

However, the extent to which such visa retaliation happens also seems to depend on the geopolitical clout of the target country. For instance, citizens from France continued to enjoy visa‐free travel to Morocco despite the fact that France also introduced visas for Moroccans, in 1986 (DEMIG VISA 2015). The existence of close diplomatic, political, and economic ties with France arguably explains why Morocco was not in the position to impose travel visas for French citizens: this could have negative repercussions for Moroccan businesses and elite interests. While diplomatic relations between Morocco and the Netherlands have always been less stable, and at times antagonistic, (Kahmann 2014), the Netherlands is also of relatively minor economic and political importance to the Moroccan state. The Moroccan state could afford to impose travel restrictions for Dutch citizens.^2^ This suggests that analyzing the evolution of reciprocal visa regimes can provide a unique window for studying power asymmetries between countries. Based on the Moroccan example, we could hypothesize that more powerful states (in terms of population, wealth and military power) have a higher ability to unilaterally impose visa conditions while securing visa‐free travel for their own citizens.

This has also been the case for many Latin American countries, which did not reciprocally impose visa requirements on Spanish citizens when visas were introduced for their citizens in 1991 amid Spain becoming part of the Schengen area. The recent openness of the Schengen area to countries including Colombia and Peru (which became visa‐exempt in 2015 and 2016, respectively) seems to reflect Spanish and European strategic economic interests in the region in the aftermath of the southern Eurozone economic crisis, which was accompanied by an increase in emigration of Spanish citizens to Latin American countries. This renewed openness was welcomed by Peruvian president Ollanta Humala, arguing that “In 1492 the first European mission arrived in America and we did not request a visa. Since that time, we have always welcomed them with open doors” (El País 2016). In sum, international visa regimes seem to mirror not only global geopolitical inequalities but also foreign relations and international alliances, either in the form of regional blocs or the existence of post‐colonial, historical, cultural, and linguistic ties.

Such inequalities and asymmetries should not necessarily be conceptualized as a global “North‐South” divide. The example of the Morocco‐France‐Netherlands triad already illustrates that realities may be more complex. This shows the need to study visa regimes at the regional and sub‐regional levels. “Southern” countries cannot be conceptualized as victims of unilaterally‐imposed visa requirements by “Northern” countries. This is also shown by the examples of many African countries, which, after independence, imposed visa regimes for citizens of former colonial powers and other foreigners. For instance, after independence in 1962, the left‐wing factions in the post‐revolutionary, socialist Algerian government tried—but ultimately failed—to impose an emigration ban to France because they saw emigration as a continuation of the colonial exploitation of labor and as detrimental to the long‐term interest of workers. Although pragmatist factions—who saw temporary emigration as a way to relieve unemployment, generate remittances, and develop workers’ skills abroad—eventually gained the upper hand, the government only accepted this point of view grudgingly (Miller 1979). This attitude must further be seen in the context of the anti‐French sentiment which followed the extremely violent Algerian war of independence (1954 to 1962).

After the Cuban revolution in 1959, the Communist government enforced strict travel visa regulations for travelers as well as exit visas for Cubans which were not lifted until 2013 (Betancourt 2014). After Suriname became independent in 1975, the Dutch government introduced a visa requirement for Surinamese citizens in 1980, which was followed—and mirrored—by a reciprocal retaliatory introduction of Surinamese entry requirements for Dutch citizens in 1982. Such measures are often associated to more general anti‐imperialist and protectionist policies, which were particularly strong in communist, socialist and “non‐aligned” former colonies. In addition, visa requirements should be seen as a practical measure aimed at controlling mobility and, hence, limiting the unwanted intrusion of foreign, colonial or capitalist influences, as well as a symbolic measure that asserted the newly‐won independence and sovereignty of a new state.

A government whose citizens are targeted by the removal or introduction of a visa requirement can react in four different ways, resulting in eight different types of bilateral visa removal or introduction dynamics (see Table 1). Besides visa retaliation as in the Surinamese–Dutch case, the second type of bilateral visa interaction is visa rapprochement. In this case, which is the opposite of visa retaliation, governments mutually lift visa requirements for each other's citizens, often, but not necessarily, as part of diplomatic agreements. For instance, Indonesia lifted travel visa requirements for Suriname (along with 74 other countries) in September 2015 and Suriname lifted entry requirements for Indonesia (along with 12 other countries) in March 2016.^3^ This “mutual gratification” was strongly related to the wish to stimulate tourism, trade and investment on both sides, but cannot be seen in isolation from broader policies of economic liberalization. In 2016, the government of Saudi Arabia indicated it would be open to visa removal for foreign citizens where their governments also lifted visas for Saudi citizens. This happened as part of a discussion of the negative economic effects of high visa restrictions in a broader context of falling oil revenues and the Saudi government's aim of diversifying and stimulating economic growth and foreign investment (Samaa 2016). These examples illustrate that visa policy dynamics need to be understood in the context of general economic policies and geopolitical shifts.

**Table 1:** Bilateral visa dynamics

		Country Y			
		Visa removal	Maintain visa‐free	Maintain visa‐restriction	Visa introduction
Country X	Visa removal	Mutual gratification	Bilateral opening	Unilateral opening	“Negative turnaround”
	Visa introduction	“Positive turnaround”	Unilateral closing	Bilateral closing	Mutual retaliation

The introduction of or lifting of visa requirements by pairs or groups of countries can thus not be seen in isolation from one another as they tend to be closely related to broader diplomatic positioning and geopolitical games. The lifting or introduction of visas does not necessarily provoke a retaliating or conciliatory reaction; in which case the measure remains unilateral. It seems safe to argue that such “unilateral visa imposition” often reflects power asymmetries, although we cannot assume that these follow a simple global North‐South divide, but rather reflect more complex, multi‐layered regional relations. A unilateral visa opening may also be followed by a visa closing, and the other way around, although this seems less common.

International visa dynamics often extend beyond the bilateral level. This is particularly important in the context of the formation of regional economic unions or “blocs.” While the EU's free migration zone and Schengen border check‐free travel zone (largely, but not entirely, overlapping with the EU) are the best‐known examples, there are currently at least 20 other regional economic unions and communities in the world (see section on *Regional free mobility clusters*), such as the Association of Southeast Asian Nations (ASEAN) free trade area, the North America Free Trade Agreement (NAFTA), the Gulf Cooperation Council (GCC), Mercado Común del Sur (MERCOSUR) and ECOWAS. Usually, but not exclusively or necessarily, such regional communities, unions, or agreements aim to liberalize trade, investment, travel, and migration among member states. Depending on the type of agreements and the degree to which these are enforced on the ground, this may coincide with a multilateral coordination of travel visa and other migration policies. For instance, an internal opening of travel and closure may go along with, or even require common external border policies. For example, when most EU countries started to remove their internal boundaries with the signature of the Schengen agreement in 1985 and its full implementation in 1995, they became increasingly concerned about controlling external borders. The suspension of internal border controls (internal opening) created an intrinsic need for common rules on travel visa requirements (external closure) and, hence, the creation of a common “Schengen visa.” This coincided with the coordinated introduction of travel visa requirements for a range of non‐European, particularly African and Asian, nationalities. Although most Asian and African nationals already needed visas to travel to Europe before, the difference was now that Schengen countries needed to align themselves by collectively deciding on which citizens needed visas to prevent them travelling into the Schengen area through countries that did not require visas. In this way, “unwanted” foreigners would be prevented from entering the entire Schengen area via the one or two countries that would not require travel visas.

This process compelled some member countries to reluctantly introduce visa requirements disrupting the long tradition of barrier‐free travel. For instance, in 1990 and 1991, Italy and Spain introduced travel visas for citizens of countries including Algeria, Morocco, Senegal, Tunisia, and Turkey as part of a move to align regulations with “European community norms” (Focus‐Migration 2012: 3; OECD 1992: 77). This move particularly upset close Moroccan‐Spanish relations. While the Moroccan government saw this as a diplomatic affront, and left the Spanish government deeply embarrassed, given the strong post‐colonial, historical, economic, and social ties between northern Moroccan and southern Spain (Zaragoza‐Cristiani 2016), the perceived benefits of joining the Schengen zone were clearly seen as outweighing the economic and political damage of border closure with Morocco.

The need to create common rules inevitably leads to a certain degree of internal horse‐trading in which countries secured visa‐free travel for citizens of countries of strategic importance. Strong Portuguese‐Brazilian relations were, for instance, likely to play a role in explaining why Brazilians have continued to enjoy visa‐free travel into the Schengen zone. In fact, given its population and size of its economy, Brazil is now a much more powerful country than Portugal. This further adds doubts to the idea that we can describe global visa dynamics in terms of a growing North‐South divide. More generally, the whole notion of “South” and “North” has been heavily criticized not only because of its colonial overtones but also because of its failure to acknowledge the huge diversity and lack of political unity within these two imaginary blocs, to the point of becoming rather meaningless.

Visa regimes are also an important negotiation chip in international relations. For instance, the fact that Turkish citizens need a visa to get into the EU, but EU citizens enjoy *de facto* visa‐free entry into Turkey, creates some pressure on the EU to lift such a requirement, particularly given Turkey's official status as an accession state.^4^ In fact, the lifting of travel visa requirements for its citizens has been one of the central long‐term goals of Turkish diplomacy, and one of the main quid pro quibus in exchange for full collaboration with the EU's border policies, and, particularly, the prevention of migrants crossing the EU's external borders illegally. It was, therefore, no surprise that the prime conditions of collaboration within the so‐called “refugee deal” of 2016 were that the EU would commit to eventually lifting travel visas for Turkish citizens in exchange for Turkish collaboration in increasing border controls and accepting the return of asylum seekers.

However, Turkey and other “labor frontier” states (Skeldon 1997) such as Morocco and Tunisia have only limited incentive to fully accede to the EU's wish to outsource or externalize migration controls and act as the EU's border guards unless these countries themselves are fully included in the greater European free mobility space through the lifting of visa requirements. After all, if illegal border crossings were to largely cease, these countries would give away an important negotiation tool to extract concessions in their own interests. This explains why these states have generally refused to sign, or to fully implement, agreements for the readmission of third country nationals—an element crucial to the success of the EU's externalization policies.

These examples suggest that the degree of bilateral reciprocity in travel visa regulations can provide a unique empirical tool with which to study migration control, international power relations, and inequalities. This is particularly important because the concept of power is so difficult to measure quantitatively. The DEMIG VISA database can therefore not only function as a proxy to measure the evolution of migration policy but, more generally, provides a powerful vantage point from which to study patterns and shifts in inter‐state power relations.

## The DEMIG VISA database and methods

Between 2010 and 2014 as part of the Determinants of International Migration (DEMIG) project at the International Migration Institute (IMI), University of Oxford^5^, we compiled the DEMIG VISA database. This database tracks visa requirements and exceptions, as well as exit permit requirements of 214 countries for travelers from 237 countries and territories over the 1973–2013 period.^6^ Our primary data are based on the regulations provided by the Travel Information Manuals (TIM) of the International Air Transport Association (IATA). The manuals published by IATA are released monthly. These manuals contain information on the travel visa requirements for travelers entering the country according to the passport they hold, as well as exit requirements for their own citizens. We have selected all manuals from January of each year^7^ to provide consistency in relation to the time at which visa changes might occur during the year.

A few other studies have already used travel visa data to study the usefulness of these policy tools and their impact on migration and border regimes (see Neumayer 2006; Hobolth, 2014; Mau et al. 2015). However, the depth and breadth of these databases are more limited than the DEMIG VISA database. For example, Hobolth (2014: 427) utilizes a European Visa Database of bilateral pairs of receiving and sending countries for the period 2005–2012, using legal documents and white papers from different European governments. Neumayer (2006: 77) takes a global approach by capturing information on visa restrictions for 189 countries taken from the TIM manuals from November 2004. Mau et al. (2015) also rely on the TIM manuals from IATA by compiling bilateral for two years: 1969 and 2010. DEMIG VISA drastically expands the coverage of previous databases by providing a year‐to‐year longitudinal bilateral travel visa database capturing the entire period between 1973 and 2013 with full global coverage. Besides entry visas, DEMIG VISA also captures exit permits^8^ which have not been previously compiled, to the best of our knowledge, by any other database.

The DEMIG VISA database captures bilateral (country‐to‐country) travel visa requirements with regards to both entry and exit requirements.^9^ Because of its full bilateral coverage, it does not introduce an artificial (and bias‐creating) distinction between origin and destination countries but treats all countries as both. The variables of the DEMIG VISA database include (1) countries reporting travel visa issuance or exit permit requirements for travelers entering and leaving the country; (2) the nationality of the travelers who are visa exempt or who require a visa; and (3) the policy measure (travel visa or exit permit). The values entered are 0 if travel visa/exit permit not needed; 1 if travel visa/exit permit needed. The value 2 is assigned if nationals of a particular country are not allowed to travel to this country of destination.^10^ We have accounted for changes in country configurations (unification and dissolution) over time by leaving respective cells blank when these countries did not yet exist or ceased to exist. These are reported as missing data. For countries with dependent territories, unless stated otherwise, we assume a visa exemption applies to all the dependent territories.^11^

Although the IATA manuals offer detailed information about the length of time travelers can stay in the country of entry, DEMIG VISA does not distinguish between travel visa exemptions for different lengths of stay. In relation to the types of passports tracked, this database only records visa and exit requirements that applied to regular travel. Hence, exemptions for diplomatic, any other official passports, or traveling for business purposes have not been recorded. We have also not considered visa exemptions for holders of residence permits in the country of visa issuance, such as for instance might apply to holders of residence permits in the Schengen area who are allowed to travel within the Schengen area.

With regards to exit permit data, we have tracked the exit regulations for both citizens of the reporting countries and foreign nationals as 0 (no exit permit required), and 1 (exit permit required). Exit permits, which entail a wide range of regulations and comparability across countries, are challenging to capture with a binary code. Exit regulations can relate to a travel clearance document acquired at the border indicating that a foreign traveler has not overstayed the entry visa, or that nationals are permitted to leave the country after being cleared by government institutions that regulate the exit of citizens. Moreover, when an exit permit is requested for foreign nationals, it is sometimes restricted to specific passports or when visitors have stayed beyond a particular period of time. As with the entry visas, we coded the exit permit variable when applied to regular passport holders, irrespective of time of validity and other specific conditions.^12^

To measure the extent to which different countries, world regions, and regional unions, have opened or closed to other countries and regions, we constructed indices of regional inbound and outbound entry visa as well as exit restrictiveness. As a first step, we calculated country‐level visa restrictiveness indices. For the inbound entry visa restrictiveness index, we computed the percentage of origin countries (all countries except the country for which the index was calculated) whose citizens need a travel visa to enter a destination country for every year.^13^ For the outbound entry visa restrictiveness index, we reversed the procedure, calculating for each country the percentage of destination countries for which its citizens would need a visa. We repeated this procedure for every year of the 1973–2013 period covered by the DEMIG VISA database. As a last step, we calculated visa indices at the levels of continents (Africa, the Americas, Asia, Europe, Oceania, as well as world regions, and other aggregates such as OECD countries) by calculating the average value of inbound and outbound visa restrictiveness for all countries within the regional aggregates for every year.

## The evolution of entry visa regimes

**Figure 1:**
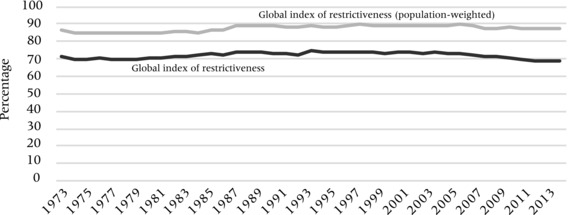
The evolution of Global visa restrictiveness NOTE: The index denotes the percentage of country‐dyads that are visa constrained, and alternatively, weighted by the size of respective populations.

Figure  1 depicts the evolution of global inbound visa restrictiveness over 1973–2013. It shows that travel visas are the rule rather than the exception: over the past four decades, less than a third of all bilateral corridors are visa‐free, that is about three‐quarters of all bilateral dyads in the world are visa‐restricted. However, the data also show that there has not been a steady increase in global trends of travel visa restrictiveness, despite common perceptions that visas barriers have been on the rise. Instead, we see a slightly hump‐shaped pattern in which the proportion of visa‐constrained dyads increased between 1980 and the mid‐1990s, but it started to slowly decrease after 2000.

Figure 1 also reports a population‐weighted index of (inbound) travel visa restrictiveness which takes into account the population sizes of both the visa‐implementing destination d and the target country o.^14^ It shows that that the level of visa restrictiveness is higher when we take population sizes into account. While approximately 30 percent of all bilateral dyads are visa‐waived corridors, only about 15 percent or less of the world population is benefiting from liberal visa policies. This implies that larger countries (such as China, India, or Nigeria) are relatively more restrictive for incoming foreign travelers and/or face entry that is more restrictive for their own citizens traveling abroad. This trend seems only to reverse slightly over the past decade.

Table 2 documents the relatively high degree of stability in global visa regimes. On average, 71.5 percent of all 1,631,701 bilateral year cases measured over the 1973–2013 period were visa‐constrained. Blacklisted corridors—where visas are systematically denied and travel is banned—are a special category, representing 3,074 visa corridor year cases, or 0.19 percent of all corridors included in the database. Examples include travel restriction for Cubans to the US, travel bans for Israelis to various countries in the world, and travel bans by Japan to nationals of the Democratic People's Republic of Korea. Over the entire period, only 1.42 percent of the visa corridors witnessed a change, of which 0.81 percent (or 13,204 cases), were a visa removal and 0.61 percent (or 9,992 cases) a visa introduction.

**Table 2:** Global visa restrictions and policy changes, 1973–2013

Dyad‐years	Frequency	Percentage
Visa‐free	462,074	28.32
Visa‐constrained	1,166,553	71.49
“Blacklisted”	3,074	0.19
Total	1,631,701	100

Visa policy changes	Frequency	Percentage
Visa removals	13,204	0.81
Visa introductions	9,992	0.61
Total change rate	23,196	1.42

NOTE: The total number of dyad‐years in the dataset (N = 2,041,718) is the product of the number of visa‐issuing reporting countries (N = 211) times the maximum number of visa‐targeted nationalities (N = 237) times number of years (N = 41). This number is corrected by some missing data. Percentage of visa policy changes is calculated as the frequency of one type of policy change divided by the total number of dyad‐years (1,631,701) in the entire dataset. The total change rate is the sum of both types of policy changes (visa removals and introductions) divided by the total number of dyad‐years.

**Figure 2:**
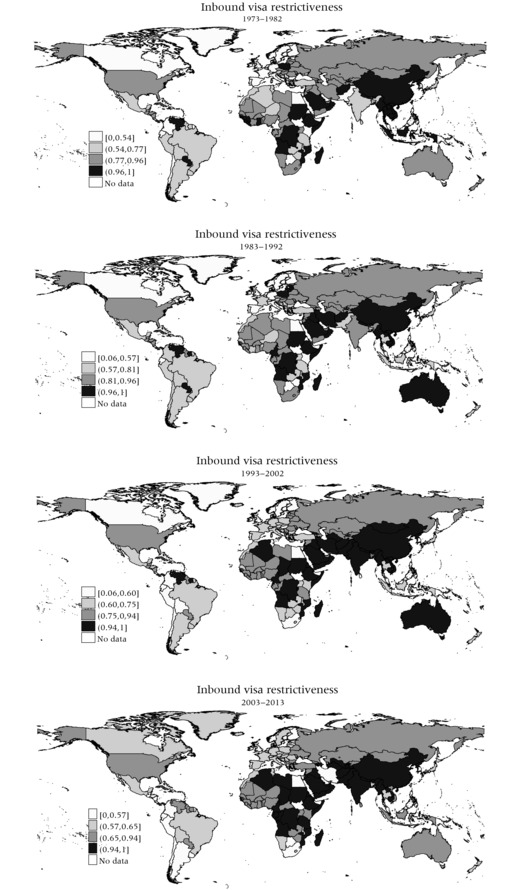
Inbound visa restrictiveness, (1) 1973–1982, (2) 1983–1992, (3) 1993–2002, (4) 2003–2013 NOTES: The four (quartile) intervals include an equal number of visa‐issuing countries of destination).Interval boundaries reflect level of (inbound) visa restrictiveness of 0th, 25th, 50th, and 75th percentile,which can and do change over time.

Figure 2 shows that the levels of inbound travel visa restrictiveness display striking variations at the regional level. Perhaps surprising, Africa turns out to be the continent with the highest levels of inbound visa restrictiveness, which has been particularly increasing in West, East, and Central Africa. Southern Africa (Namibia, Botswana, South Africa) is the only region in which levels of visa restrictiveness have decreased since the 1990s, which seems to be related to the end of the *apartheid* regime, the end of wars of liberation from colonial rule, and the relaxation of previously‐strained relations with the Frontline States (Angola, Botswana, Mozambique, Tanzania, Zambia, and Zimbabwe). North Africa shows a mixed pattern, with Libya and Algeria showing increasing restrictions, and Morocco and Egypt maintaining stable, comparatively liberal inbound travel visa regimes. Also, countries in the Middle East and large parts of Asia (excepting a few countries including Bangladesh, Thailand, Malaysia, and the Philippines) tend to have highly restrictive visa regimes, and levels have increased rather than decreased, particularly in Iraq, Pakistan, and India. Some countries show non‐linear trends, like Indonesia, where levels of visa restrictiveness first decreased and then increased. Iran stands out for a sudden and striking drop in restrictiveness during the decade between 2003 and 2013. Visa restrictiveness levels have been consistently high in countries such as China, Vietnam, Laos, Cambodia, Myanmar, and Nepal.

Inbound visa restrictiveness in Russia and other Commonwealth of Independent States (CIS) countries has remained stable at moderately high levels, although visa restrictions have further tightened in a few countries such as Turkmenistan, Uzbekistan, and Tajikistan. Europe shows a mixed picture, more or less along the lines of the former East‐West divide. During the 1970s, Western Europe had some of the lowest levels of visa restrictiveness in the world (also compared to other liberal democracies such as the US); levels have increased since the 1980s to moderate but still relatively low levels. However, for Eastern Europe, the trend has rather been the opposite, particularly in new EU accession states like Poland and Romania, where the end of communist rule coincided with a decrease in visa restrictiveness.

In Latin America, travel visa regulations have become even more liberal over recent decades, particularly in some Andean countries (Colombia, Venezuela, Ecuador), as well as other countries in South and Central America (such as Chile). This coincides with the end of authoritarian regimes in the region, which has led to a relaxation of visa rules, as well as the opening up to neighboring countries. Some countries show non‐linear trends. For post‐independence Suriname, for instance, visa restrictiveness first increased and then decreased. The US has had rather consistent levels of moderately high visa restrictiveness, while Canada shows an increase from low to moderately‐low levels in the 2000s. In Australia, levels of restrictiveness first increased, but then dropped over the 2000s, while New Zealand has shown a liberalizing trend.

**Figure 3:**
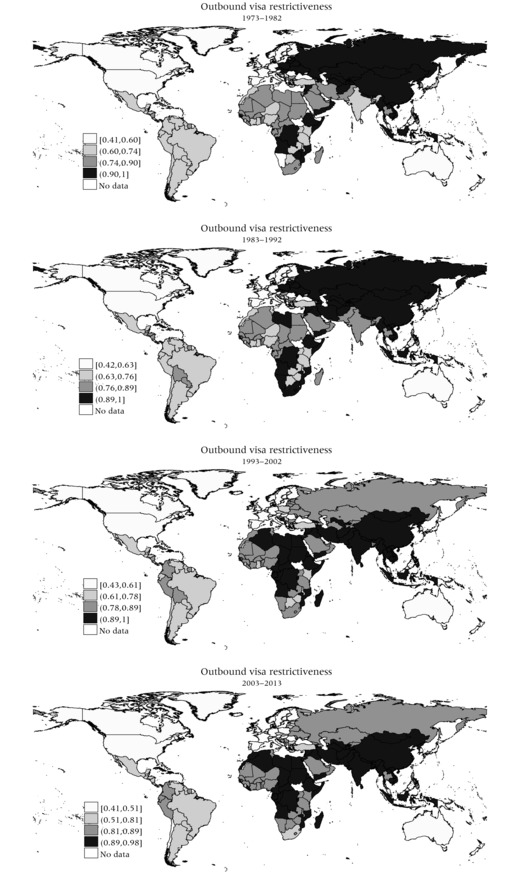
Outbound visa restrictiveness (1) 1973–1982, (2) 1983–1992, (3) 1993–2002, and (4) 2003–2013 NOTE: The four (quartile) intervals include an equal number of visa‐targeted countries (of origin). Intervalboundaries reflect level of (outbound) visa restrictiveness of 0th, 25th. 50th, and 75th percentile, which canand do change over time.

Turning the perspective around, Figure 3 portrays the levels of outbound travel visa restrictiveness, which measures the degree to which citizens of each country require visas to enter other countries. This yields a more clear‐cut pattern compared to those of inbound visa restrictiveness. Clearly, citizens of Western Europe and the former Western‐European settler colonies of the US, Canada, Australia, and New Zealand have the highest degree of visa‐free travel liberties. While citizens of the former Soviet Union and formerly communist Eastern European countries used to face high levels of outbound visa restrictiveness, these levels have receded remarkably since the fall of the Berlin Wall and the dissolution of the Soviet Union, particularly in new Eastern European EU member states and also in Russia. Also, Latin Americans face comparatively low visa barriers to enter other countries, and trends have been downward, particularly for Argentina, Brazil, and Chile and several Central American countries, although they have been high or increasing in Colombia and the former British and Dutch colonies of Guyana and Suriname.

By contrast, levels of outbound travel visa restrictiveness have been comparatively high in Africa and clearly increasing in most countries that enjoyed relatively high degrees of visa‐free travel opportunities in the 1970s, such as Kenya, Uganda, Namibia, Botswana, and Tunisia, and to a certain degree also countries such as Morocco, Egypt, Ghana, and Senegal. South Africa is one of the few exceptions where visa‐restrictiveness for travel abroad has decreased. Also, for countries in the Middle East, including Turkey and Iran, outbound travel restrictiveness has clearly increased. This restrictiveness has also been consistently high for most South, East, and Southeast Asian countries, despite a few exceptions such as Japan, South Korea, and Malaysia. Outbound visa restrictiveness for Chinese citizens has been consistently high, while for India and Pakistan levels have been increasing.

Travel visa reciprocity has been relatively stable over the last four decades at rather high levels, as it has only fluctuated between 78 and 81 percent over the entire period.^15^ Nevertheless, since the mid‐1990s, we see a trend toward higher levels of visa reciprocity under which visa waivers or visa restrictions mirror each other on either side of migration corridors (see Table 1). This is likely to reflect geopolitical shifts after the end of the Cold War and a “re‐sorting” of foreign policy priorities and relations as well as processes of regionalization, such as the enlargement of the EU. Asymmetric, non‐reciprocal bilateral travel visa policies are often only temporary as either retaliation or gratification is the most common response to a (positive or negative) visa policy change. But in some cases, bilateral corridors remain open only in one direction and it is mostly to the disadvantage of those countries that are in an economically and/or politically weaker and thus more dependent position.

## Regionalization of travel visa policy priorities: patterns of closure and opening

So far, the analysis has shown that, although citizens of poorer regions generally face greater barriers to travel abroad than citizens of European countries and former countries of colonial European settlement, the degree of variation is rather high, and trends over time on regional and country level can vary considerably and be divergent. The contrast between trends in Latin America on the one hand and Africa and the Middle East on the other is particularly remarkable. We have also seen that there is no clear‐cut relation between levels of inbound and outbound restrictiveness. For instance, while most Latin American countries have low levels of inbound and outbound restrictiveness, sub‐Saharan Africa and some Asian countries like China have very high levels on both measures. While Iranians require visas for the vast majority of countries, inbound restrictiveness is relatively low. Europeans, Brazilians, Indians, and Saudis face fewer visa obstacles when traveling abroad than do visitors to their own countries.

**Figure 4:**
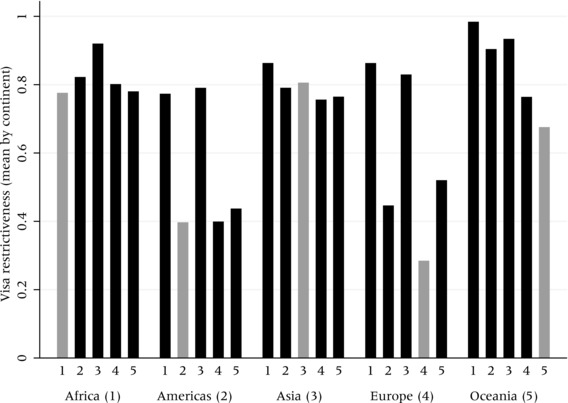
Index of visa restrictiveness within and between continents NOTES: Gray bars show intra‐regional visa restrictiveness whereas black bars show respective rates againstnationals of other continents. Index represents the share of all country dyads—either within or outside aworld region—which were visa‐constrained between 1973 and 2013.

To investigate the degree to which world regions are relatively open or closed vis‐à‐vis other world regions, Figure 4 plots average levels of inter‐ and intra‐continental visa restrictiveness over the entire 1973–2013 period. It shows some striking patterns. For instance, Africa and Asia appear to be the most restrictive regions in the world with regard to the restrictions they impose on travelers from other world regions as well as from their own regions. Countries in the Americas impose high restrictions for Africans and Asians, but relatively low restrictions for other Americans and Europeans, which seems to clearly reflect their immigration histories. We find similar patterns for European countries. Intra‐European travel is the least visa‐restricted in the world, which largely reflects EU integration and enlargement.

**Figure 5:**
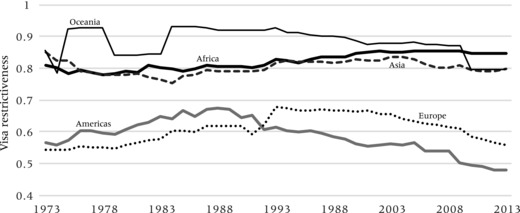
Index of inbound visa restrictiveness, 1973−2013 by continent of destination NOTE: Index represents the share of all countries of a world region which have been requesting travel visas from any nationals.

**Figure 6:**
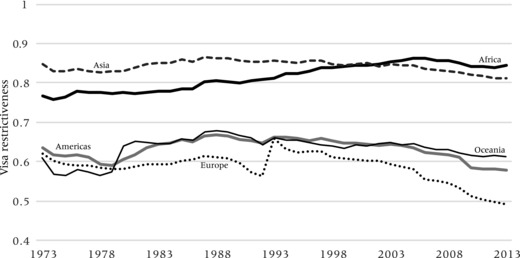
Index of outbound visa restrictiveness, 1973−2013, by continent of origin NOTE: Index represents the share of all nationals of a world region which require a travel visa for entering another country.

Figures 5 and 6 show how the levels of inbound and outbound visa restrictiveness evolved over the 1973–2013 period. They highlight the overall finding that visa regimes have been rather restrictive and stable. Figure 5 confirms that levels of inbound restrictiveness are highest and comparatively stable in Africa, Asia, and Oceania. For the Americas and Europe, these are significantly lower, and show an increasing trend up to the mid‐1990s, after which they have started to decrease. This overall trend cannot solely be explained by the fall of the Berlin Wall and/or EU enlargement, but also by the striking liberalization of Latin American visa regimes. Unsurprising, levels of outbound restrictiveness are highest for Asians and Africans, where they have also been increasing for Africans. Visa barriers are lower for citizens from other world regions and, particularly for Europeans, have been decreasing.

The results confirm the overall patterns observed so far, but also show significant intra‐regional variation.^16^ For the 2000s, within Africa, particularly Western (88 percent) and Central Africa (93 percent) have extremely high levels of inbound visa restrictiveness. Within Oceania, Melanesia (86 percent) shows high levels of restrictiveness, whereas those levels are lower in Australia and New Zealand. Two very different regions, Northern Europe and the Caribbean, are the regions with the lowest levels of inbound restrictiveness, of 42 percent and 45 percent, respectively. Northern Europeans and North Americans have the lowest levels of outbound visa restrictiveness at levels of 45 percent and 44 percent respectively. North and Central Africans and South Asians face the highest travel visa barriers with restrictiveness levels hovering around 91–92 percent. In terms of the evolution of visa regimes over time, we see varying trends, with some regions showing linear trends toward opening or closure and other regions showing non‐linear trends, such as Southern Asia, Central America, and Eastern Europe becoming more restrictive initially and liberalizing in more recent decades.

Table 3 displays a typology of four categories combining inbound and outbound travel restrictiveness for 20 world regions. Most “Western” world regions, including the Caribbean, are characterized by relatively low overall levels of visa restrictiveness, with levels of both inbound and outbound visa restrictiveness significantly below global averages. While various African and Asian regions “respond” to high levels of outbound restrictiveness with equally restrictive levels of inbound policies, South and Central American regions are rather characterized by high levels of inbound restrictiveness (mostly toward non‐Western citizens) while enjoying relatively low levels of outbound restrictiveness. Finally, citizens of North and East Africa, South‐East and South Asia face relatively high visa obstacles to travelling abroad, but these regions are comparatively open to the inbound visa‐free travel of foreigners.

**Table 3:** Typology of regional visa regimes

		Outbound restrictiveness	
		Low	High
Inbound restrictiveness	Low	Caribbean	Eastern Asia
		Northern Europe	Eastern Africa
		Western Europe	South‐Eastern Asia
		Northern America	Northern Africa
		Southern Europe	Southern Asia
	High	Australia and New Zealand	Western Africa
		South America	Western Asia
		Central America	Central Asia
		Southern Africa	Middle Africa
		Eastern Europe	Melanesia

NOTE: Cut‐off points between low and high levels of restrictiveness are about 70 percent of inbound restrictiveness and 80 percent of outbound restrictiveness. These cut‐off points represent global medians on both indicators.

**Figure 7:**
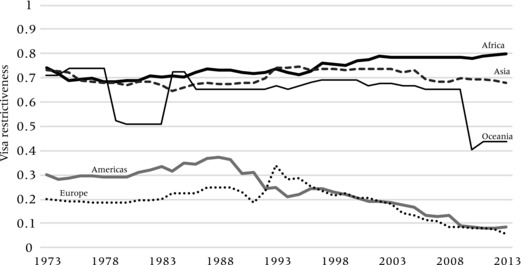
Index of outbound visa restrictiveness toward OECD citizens, 1973−2013 by continent of destination NOTE: Index represents the share of all nationals from an OECD country which require a travel visa for entering another country.

**Figure 8:**
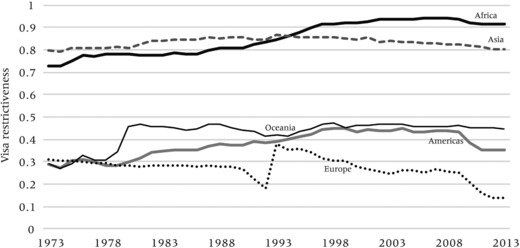
Index of inbound visa restrictiveness of OECD countries toward foreign citizens, 1973−2013, by continent of origin NOTE: Index represents the share of all OECD countries that request a travel visa from any national.

Further challenging the idea that there is a clear‐cut “global mobility divide” between citizens of comparatively wealthy and poor countries, Figures 7 and 8 show trends of visa restrictiveness for citizens of OECD countries taken together. Figure 7 shows that OECD citizens barely need visas to travel to either the Americas or Europe, with restrictiveness levels dropping from an already low level of 20–30 percent in 1973 to below 10 percent in 2013. This reflects the fact that OECD countries have removed visa barriers amongst each other in the first place. At the same time, visa‐constrained entry of OECD citizens into African and Asian countries has remained high at levels of at least 65 percent, while this level has been even increasing, particularly in Africa, reaching levels of around 80 percent. Consequently, the assumption that citizens of wealthy countries can travel freely over the world is flawed as suggested in the introduction of this paper, although one can say that they can travel freely to most countries they tend to travel to.

Figure 8 assesses the evolution of the level of visa constraints for people traveling to OECD countries. It replicates earlier findings that Asians and Africans face the highest constraints of entering OECD countries. Trends for Asians have been relatively stable (varying around 82 percent). Trends for African citizens are slightly hump‐shaped, with visa‐restrictiveness levels increasing from 73 percent in 1973 before plateauing at levels of around 93 percent in the early 1990s, with only a minor decrease since 2010. As expected, Europeans and (North and South) Americans have the greatest ease of travel to (other) OECD countries, reflecting the idea that the visa liberalization has been to a significant extent an intra‐American, intra‐European and intra‐OECD affair. In fact, citizens from most Asian and African countries have remained excluded from free travel at comparatively high levels.

## Regional free mobility clusters

**Figure 9:**
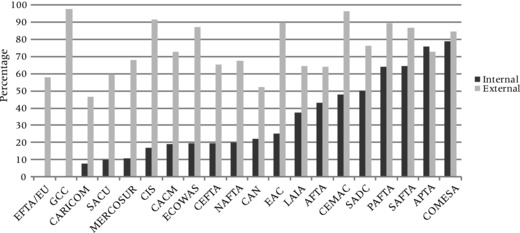
Internal versus external inbound visa restrictiveness of regional economic blocs NOTE: AFTA: ASEAN Free Trade Area; APTA: Asia Pacific Trade Agreement; CACM: Central American Common Market; CAN: Andean Community; CARICOM: Caribbean Community; CEFTA: Central European Free Trade Agreement; CEMAC: Economic and Monetary Community of Central Africa; CIS: Commonwealth of Independent States Free Trade Agreement; COMESA: Common Market for Eastern and Southern Africa; EAC: East African Community; ECOWAS: Economic Community of West African States; EFTA: European Free Trade Association; EU: European Union; GCC: Gulf Cooperation Council; LAIA: Latin American Integration Association; MERCOSUR: Mercado Común del Sur — Common market of the South; NAFTA: North American Free Trade Agreement; PAFTA: Pan‐Arab Free Trade Area; SACU: Southern African Customs Union; SADC:Southern African Development Community; SAFTA: South Asian Free Trade Agreement.

To analyze visa policy dynamics at the regional level, Figure 9 compares average levels of *internal* and *external* inbound visa restrictiveness for 20 regional economic blocs. Internal visa restrictiveness measures the extent to which citizens from other member countries need travel visas, whereas external visa restrictiveness applies to visa requirements for citizens from non‐member or so‐called “third” countries. The most “integrated” regional unions in terms of implementation of visa‐free travel are the EU/European Free Trade Association (EFTA) and the GCC, but also the Caribbean Community (CARICOM), the Southern African Customs Union (SACU), MERCOSUR, CIS, the Central American Common Market (CACM), ECOWAS and the Central European Free Trade Agreement (CEFTA) have levels of internal visa restrictiveness below 20 percent. By contrast, other regional economic communities including the Pan‐Arab Free Trade Area (PAFTA), the South Asian Free Trade Agreement (SAFTA), the Asia Pacific Trade Agreement (APTA) or the Common Market for Eastern and Southern Africa (COMESA) show still very high levels of both internal *and* external travel restrictiveness.

**Figure 10:**
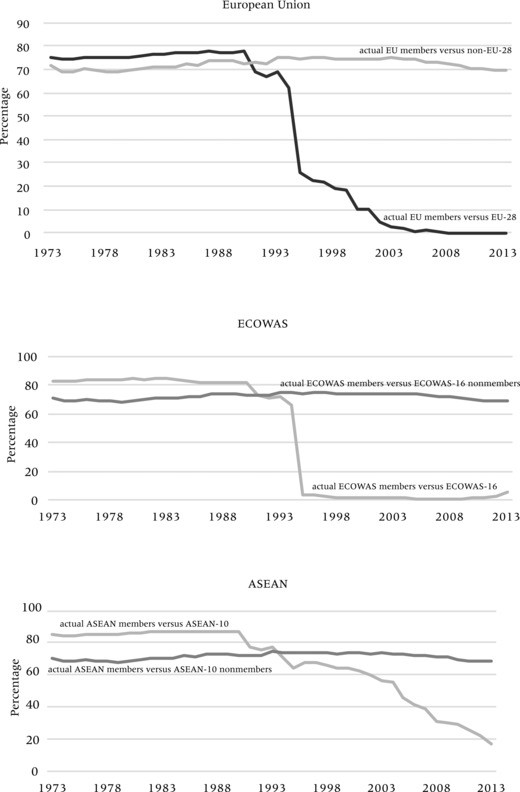
Regional integration and internal versus external visa policy changes (visa restrictiveness index)

To further investigate the evolution of the interrelated processes of internal opening and external closure of regional blocs, we have focused on the dynamics in the EU, ASEAN, and ECOWAS regions. Figure 10 shows how internal visa openness among member states does not necessarily coincide with an increasing external closure towards non‐members, mainly because this external closure was already very high. Thus, regional integration is mainly expressed through an internal opening through free travel while the external closure is maintained at high levels. For example, the case of the EU shows that inbound visa restrictiveness toward citizens of non‐member states have remained relatively stable over time, both before and after EU enlargement and an internal opening. A similar pattern occurs for ECOWAS (although with a more sudden shift in openness for its members than in the case of the EU, where enlargement was a more stretched‐out process); and for ASEAN, where external closure has not further increased with internal opening but has rather been maintained at high levels.

The idea that global visa regimes, instead of following a dichotomous global North‐South mobility divide, should rather be conceptualized as a pattern of partly geographically separate (e.g. GCC, ECOWAS), partly geographically overlapping (e.g. EU with OECD), regional clusters—where internal opening coexists with, and is partly functionally contingent on, external closure.

## Evolutions and patterns of exit control

As de Haas and Vezzoli (2011) argued, the regulation of exit has become less frequent over the past few decades. When states move toward more democratic forms of governments and more liberal economic systems and liberalize labor market polices, they also become less willing and able (mainly in terms of the legal limits put on executive power (Joppke and Guiraudon (2003) for a parallel case on the legal limits on immigration controls) to exert direct control of movement on their own citizens. Besides the difficulty of legitimizing exit controls in more democratic political systems, Bourguignon (1977) argued that this shift coincided with a growing conviction that immigration policies are much more effective in regulating population movement than emigration policies. Democratization and a concomitant greater respect for the human rights of their own citizens is another important factor explaining the declining powers of states to directly control emigration. Autocratic states have generally a higher ability to control emigration, and they have historically tended to impose exit controls if they have closed economic systems and wish to protect the country from foreign economic or political influences. Starting in the 1920s, emerging Fascist and Communist states, therefore, introduced policies to regulate exit (Dowty 1987; McKenzie 2005). This is why countries such as those of the former Soviet Union, China, Egypt (under the Arab nationalist socialist regime of Nasser), and Cuba have traditionally imposed exit visas. Authoritarian regimes with liberalized capitalist economic systems (such as the Philippines under the Marcos dictatorship, or Fascist Italy under Mussolini (Cometti 1958), or a Western‐realigned Egypt (under Sadat and Mubarak) tend to be more ambiguous toward emigration and may even stimulate emigration as a political‐economic “safety valve,” diminishing the pressure for political reforms (see de Haas 2012; Gammage 2006; Kireyev 2006). Nationalism and a determination to shed foreign influence partly explain why newly‐decolonized countries in Africa such as Algeria often tried to curtail emigration (and immigration) after they gained independence.

**Figure 11:**
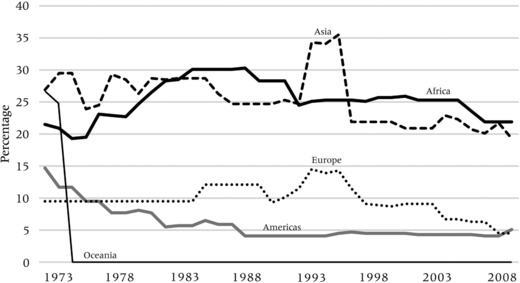
Evolution of exit restrictiveness, 1973−2008, by continent

Nowadays, only a limited number of generally authoritarian states are positioned to impose exit restrictions. For liberal democracies, the restriction of the freedom to exit your own country of citizenship is seen as an infringement of fundamental human and constitutional rights. As part of a general process of liberalization and (partial) democratization, many countries that used to impose exit restrictions have been progressively lifting these requirements over the past 40 years. Not surprising, exit restrictiveness is very low in Europe, Oceania, and the Americas, where liberal states have lifted the regulations to exit. The sharper decrease of exit restrictiveness in the Americas compared to Europe reflects the liberalization of exit controls in South and Central America during the 1980s and 1990s. As tends to be the case with entry visa restrictions, African and Asian states impose exit restrictions relatively more frequently, although also in these regions there has been a steady reduction in exit restrictiveness since the mid‐1990s (Figure 11).

**Figure 12:**
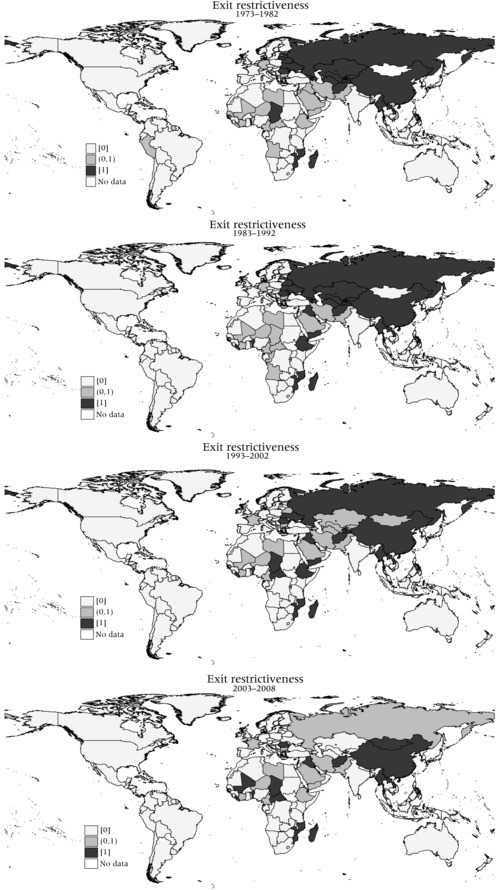
Exit restrictiveness: (1) 1973−1982, (2) 1983−1992, (3) 1993−2002, (4) 2003−2008

Figure 12 illustrates geographical and temporal variations of exit restrictions. The data show how from 1972 to 1983, most countries in the Americas and Europe did not impose any exit restrictions, with the USSR being the main exception in Europe. In the Americas, several countries including Bolivia, Brazil, the Dominican Republic, and Haiti imposed exit restrictions on their own citizens, while El Salvador and Peru selectively regulated the exit of travelers of various nationalities. Nicaragua controlled predominantly the exit of nationals of USSR and other Warsaw Pact countries, while Cuba controlled the exit of citizens and all foreign nationals (reported until 1975).

In the 1970s, Asian countries had the highest level of exit restrictiveness in the world. At that time, countries such as Afghanistan, China, Jordan, Kuwait, Lao People's Democratic Republic, Myanmar, Iraq, Iran, and Pakistan restricted the exit of most foreign nationals. Until the late 1970s, Bangladesh regulated the exit of foreign nationals from several countries, and Sri Lanka only regulated the exit of its own nationals. In the 1970s, African countries such as the Central African Republic, Chad, Côte d'Ivoire, Liberia, Libya, Mozambique, Niger and Cameroon also had high and increasing levels of exit restrictiveness for many foreign travelers. These restrictions might relate to the domestic issues which are beyond the scope of this paper related to post‐colonial wars, stabilization of the region, and the control of openness to internationalization processes. Other countries such as Sudan and Tanzania only imposed exit restrictions on their own citizens. As with the regulation of entry visas, geopolitical shifts and processes of liberalization and, to some extent, democratization in these different countries led to a gradual lifting of their exit controls, although this has not been a uniform development, with some countries moving in the opposite direction. This defies the idea of a general abolition of exit restrictions and shows that such trends can also be reversed, depending on the autocratic or democratic nature of governments. We can see on the map how in the 1980s, exit restrictions were progressively lifted in many countries, but we also see that other countries which did not require exit permits in the 1970s started to regulate exit. For example, Angola started imposing exit restrictions for most nationals from 1980 to the early 1990s, which might relate to the ongoing war in the country until the peace process in the 1990s. In this period, Asian countries experienced an exit liberalization wave, with countries such as Jordan lifting exit permits also for their own citizens. In the 1990s and 2000s, exit restrictions stabilized at generally lower levels of restrictiveness, with only a few exceptions in Europe. Most countries that still impose exit restrictions are located in Asia (such as Afghanistan and China, with requirements tracked in the IATA manuals until the mid‐2000s), Africa (such as Benin, Cameroon, the Central African Republic, Chad, Lebanon, Liberia, and Niger), and Cuba in the Americas (until 2013).

## Conclusion

Drawing on DEMIG VISA, which covers global bilateral travel regulations from 1973 to 2013, this paper investigates patterns and trends in international visa regimes. In addition, DEMIG VISA provides a lens through which to study and measure the evolution of complex, multi‐layered power inequalities and symmetrical and asymmetrical alliances between states as well as the dissolution and formation of states and regional blocs. Openness and closure of mobility through travel restrictions do not only reflect the direction of migration policy regulations but also broader shifts in bilateral and multilateral foreign policy relations. For instance, the paper has shown that the degree of visa reciprocity (the degree to which state X can impose a visa requirement on citizens from state Y without state X having to fear a retaliating measure by state Y for their own citizens) can be a valuable indicator of power asymmetries. This allows us to go beyond simplified, reductionist accounts according to which there would a global “North‐South” divide and instead enables us to map continuities and discontinuities in inter‐state relations as well as to study processes of globalization, regional opening, and external closure.

While the clearest trend has been an increased lifting of exit restrictions, levels of entry visa restrictiveness have remained strikingly stable at high levels of around 73 percent. This challenges popular ideas that states have *increasingly* closed their borders over the past decades: visas have been the rule rather than the exception. While predominantly European and North American OECD countries maintain high levels of entry visa restrictiveness for citizens from regions like Africa and Asia—which represents a stable rather than increasing level of restrictiveness—the latter regions have the highest overall levels of entry restrictions. Although citizens of wealthy countries generally enjoy the greatest visa‐free travel opportunities, this primarily reflects their freedom to travel to other OECD countries. The analysis highlights that visa‐free travel is mostly realized between geographically‐contiguous countries of integrated regional blocs such as the EU. Such intra‐regional opening up also explains the slightly decreasing trend of global visa restrictiveness since the 2000s.

Analyses of global dynamics in visa reciprocity show that a significant proportion of the country dyads have asymmetrical visa rules (e.g. France‐Senegal or Cameroon‐Botswana) but also show that levels of reciprocity have increased since the mid‐1990s, which seems to mainly reflect the formation of regional free‐travel blocs. We have argued that regional blocs have formed clusters of visa openness and external closure to satisfy requirements that privilege citizens of the regional group for internal mobility. Visa‐free travel is primarily realized within regional blocs as well as amongst OECD countries. When looking at times when opening‐closure dynamics take place, we have observed that entry regulations might have been established before the consolidation of the blocs reflecting an already high level of similarity in geopolitical and economic interests. The paper also highlights the decreasing popularity of exit permits in the last decades, particularly for Europe and the Americas, although this trend has not been linear, and the quality of exit permit data is less consistent than entry visa data.

The analysis challenges simplistic narratives according to which the Global North has increasingly closed its borders towards citizens of the Global South. First, levels of visa restrictiveness were already high back in the 1970s, and the general pattern has been one of stabilization and slight decrease rather than increase, with the exception of parts of Africa. Second, the analysis showed that regional integration is primarily manifested through internal liberalization of visa regimes, and not through external closure, as visa restrictiveness was already high previously. This challenges the idea that internal closure has coincided with increasing external closure. Third, developing countries in sub‐Saharan Africa and South and South‐East Asia are among the nations with the highest levels of inbound visa restrictiveness of the world. Thus, visa liberalization primarily reflects the integration and/or enlargement of regional blocs.

All of this exposes the deceptive nature of the North‐South framing of debates around mobility and migration, according to which the Global North closes its doors to the Global South. It corroborates more general criticism of the meaningfulness of the “North” and “South” categories (see also Bakewell, 2009). The analysis contradicts the common perception that overall levels of travel and visa restrictiveness are increasing. At the global level, visa restrictions are rather slightly decreasing. Yet, our most important observation remains that international configuration of visa regimes is primarily realized as part of regional alliances and the formation of regional blocs. Instead of a global mobility divide, it is therefore perhaps more appropriate to speak of multiple regional mobility divides in an increasingly multi‐polar world.

## Notes

1 The compilation of DEMIG VISA coincided with other efforts to collect migration policy data, such as Mayda and Patel's (2014) database of migration policies for 14 OECD countries for the period 1980–2000, its subsequent extension (until 2006) by Ortega and Peri (2012), and the Immigration Policy and Law Analysis (IMPALA) which covers 25 countries over the 1960–2010 period (Gest et al., 2014) (for a review of these and other migration data compilations, see de Haas et al. 2015). Organizations such as the UN, OECD, and the World Bank have compiled flow and stock migration data (see Parsons et al. (2005) and Özden et al. (2011)) (for an extensive review of current advancements to migration flow and stock data (see Vezzoli et al. 2014). Besides DEMIG VISA, the DEMIG project compiled two major databases on migration flows (DEMIG C2C) and migration policy changes (DEMIG POLICY). DEMIG C2C (country‐to‐country) is a bilateral migration flow database from 1946 to 2011 for 34 reporting countries. DEMIG POLICY tracks over 6,000 policy changes in 45 countries from 1946 to 2014 (see de Haas et al. 2015).

2 The formal visa requirement for Dutch citizens was lifted in 1996.

3 Source: Personal communication with Simona Vezzoli, University of Amsterdam.

4 Nominally, visas are required to enter Turkey for EU nationals, but these can be granted by paying a fee at the Turkish border with minimal inconvenience, and the process does not involve any security checks.

5 Please note: https://www.imi.ox.ac.uk/about/Note_to_our_users

6 The database is not symmetrical given that it provides data on 214 countries in total and 237 nationalities of the travellers over the period of 1973 to 2013. These fields include countries that no longer exist (e.g. USSR post‐1992), as well as dependent territories (e.g. French Guiana, Guadeloupe, or New Caledonia for France). This has generated missing values for visa requirements of countries/passports that no longer exist.

7 Except the December 2003 manual, which is used instead of the January 2004 manual as the latter was not accessible.

8 IATA defines exit permit as the permit entitling the holder to leave a country.

9 Note that the DEMIG VISA Database only tracks travel visa and exit regulations. It does not include information regarding any other type of visa (e.g. student, seasonal worker, family reunification, permanent residency, etc.).

10 Note that such travel bans only apply to entry visa of so‐called “blacklisted” nationalities.

11 For example, if a nationality is visa‐exempted for New Zealand, we have assumed this is also the case for Niue and Tokelau, which are New Zealand dependent territories.

12 The IATA manuals stopped reporting the section on exit permits in the mid‐2000s (with some level of variation for some countries).

13
Visadtinbound=1−∑i=1Ovisa_freeodt/∑i=1Ovisaodt, with d∈D={1,…,211} and o∈O={1,…,237} Visaotoutbound=1−∑i=1Dvisa_freeodt/∑i=1Dvisaodt, with d∈D={1,…,211} and o∈O={1,…,237}

14 Calculation of population‐weighted index of visa restrictiveness:$\;Visa_{odt}^{weighted}=\;1-\frac{\sum_{o}\sum_{d}Visa\_free_{odt}*Pop_{ot}*Pop_{dt}}{\sum_{o}\sum_{d}Pop_{ot}*Pop_{dt}}$

15 Visa reciprocity is defined as:
Visareprocityt=∑visaodtOxD|ifvisaodt=visadot=1+∑(1−visaodt)OxD|ifvisaodt=visadot=0; with o∈O={1,…,237} and d∈D={1,…,211}.

16 IATA's reporting system on exit restrictions is not without inconsistencies over time and also across countries, as for instance no exit restrictions are reported for Poland and Czechoslovakia before 1990 or Cuba after 1975. We retain this information for these countries. For Germany before 1990, Figure 11 shows exit restrictions implemented by the German Democratic Republic.
